# Allantoin as an independent marker associated with carotid intima-media thickness in subclinical atherosclerosis

**DOI:** 10.1590/1414-431X20187543

**Published:** 2018-06-18

**Authors:** M.S. Santana, K.P. Nascimento, P.A. Lotufo, I.M. Benseñor, F.C. Meotti

**Affiliations:** 1 Departamento de Bioquímica, Instituto de Química, Universidade de São Paulo, São Paulo, SP, Brasil; 2 Centro de Pesquisa Clínica e Epidemiológica, Hospital Universitário, Universidade de São Paulo, São Paulo, SP, Brasil; 3 Departamento de Medicina, Escola de Medicina, Universidade de São Paulo, São Paulo, SP, Brasil

**Keywords:** Allantoin, Uric acid, Common carotid intima-media thickness, Atherosclerosis, Oxidation

## Abstract

Allantoin is the main product of uric acid oxidation and was found to be augmented in atherosclerotic plaque in human autopsy and in animal models of atherosclerosis. Uric acid is abundant in human plasma and is prone to oxidation in inflammatory conditions such as atherosclerosis. In this study, we found a significant increase in plasma uric acid (P=0.002) and allantoin (P=0.025) in participants of the Brazilian Longitudinal Study of Adult Health (ELSA-Brasil) that presented common carotid intima-media thickness (c-IMT) within the 75th percentile (c-IMT≥P75). Multiple linear regression showed an association of c-IMT with uric acid (β=0.0004, P=0.014) and allantoin (β=0.018, P=0.008). This association was independent of age, the traditional risk factor LDL/HDL ratio, and non-traditional risk factors: pulse pressure, neck circumference, and the inflammatory marker myeloperoxidase. The independent and strong association of allantoin with c-IMT shows that it might be a useful marker, along with other traditional risk factors, to evaluate an early stage of atherosclerosis.

## Introduction

Increased levels of serum uric acid have been suggested as a possible predictor for cardiovascular disease (CVD) (1,2). Epidemiological studies have demonstrated a link between hyperuricemia (serum uric acid >7 mg/dL) and hypertension, kidney disease, and other risk factors for CVD, like obesity and metabolic syndrome (2,3). Serum uric acid has been associated with carotid intima-media thickness (c-IMT) and coronary artery disease (4,5). However, debate continues whether hyperuricemia predicts CVD risk independently or merely reinforces classical CVD risk factors (2,6). The increased level of uric acid in atherosclerosis has been suggested as a defense mechanism against oxidative stress because uric acid is an important serum antioxidant (7). In fact, uric acid is the main substrate for oxidation by peroxidases in plasma (8). The oxidation of uric acid by peroxidases or by oxidants yielded during inflammation generates allantoin as the main final product (9,10). Therefore, the measurement of allantoin rather than uric acid alone could indicate vascular damage related to inflammation and oxidative stress in atherosclerosis (9,11).

Substantial amount of allantoin has been found in human atherosclerotic plaques (12,13). Importantly, the degree of atherosclerosis in knockout mice prone to atherosclerosis was positively associated with urinary excretion of allantoin (14). In spite of the evidence, clinical studies that correlate plasma allantoin levels and atherosclerosis are scarce. Allantoin could be a true marker for atherosclerosis especially in the initial steps of the process when other risk factors still have weak associations. In this study, we investigated the association of uric acid and allantoin with c-IMT. We found that both metabolites were significantly associated with c-IMT independently of classical risk factors of atherosclerosis.

## Material and Methods

### Study population

A total of 35 men with c-IMT scored within the 75th percentile (c-IMT ≥P75) and another 35 men with c-IMT below the 75th percentile (c-IMT <P75), all apparently healthy, were randomly selected from the serum bank of the Brazilian Longitudinal Study for Adult Health (ELSA-Brasil) project. None presented a c-IMT higher than 1 mm to ensure the inclusion of subclinical atherosclerosis cases only. Groups were paired according to race and diagnosis of hypertension and diabetes.

ELSA-Brasil is a multicenter cohort study of 15,105 living in 6 Brazilian cities (São Paulo, Rio de Janeiro, Porto Alegre, Belo Horizonte, Vitoria, and Salvador), aimed at investigating biological and social determinants of cardiovascular diseases and diabetes. During baseline evaluation (2008–2010), the diagnoses of hypertension, diabetes, dyslipidemia, and regular use of medicines were confirmed, uric acid was measured, and the common c-IMT was determined. Subjects under use of uricosuric drugs, with body mass index ≥30, glomerular filtration rate ≤70 mL/min were not eligible for randomization. The study conformed to the Declaration of Helsinki. All participants agreed to participate in the study and signed an informed consent form. The study was approved by the Institutional Review Board of the Universidade de São Paulo (Brazil). All samples were analyzed by the double-blinded method.

### Laboratory analysis

Heparinized plasma samples were collected and frozen at -80°C. Details about proceedings at ELSA-Brasil central laboratory and biobank are described in detail elsewhere (15).

### Quantification of plasma uric acid and allantoin

Uric acid was extracted from 200 µL plasma, mixing with 600 µL of cold acetonitrile. The samples were centrifuged at 22,000 *g* for 15 min at 4°C, filtered and injected onto a high-performance liquid chromatography (HPLC, Shimadzu, Japan) equipped with two LC-20AT pumps, one auto-sampler SIL-20AC HT, absorbance detector SPD-M20A, and a CBM-20A system controller. Samples were separated in a hydrophilic interaction chromatography column TSK gel Amide-80 (150×4.6 mm, 3 µM, Tosoh Bioscience, Japan). The mobile phase consisted of 40% ammonium acetate (10 mM, pH 6.8) and 60% acetonitrile isocratic, and a flow of 0.4 mL/min. Quantification was obtained by plotting the peak area against a standard curve of uric acid (20–1500 µM). Standard solutions (10 mM) were prepared in 20 mM NaOH. This standard solution was diluted in the mobile phase just before injection. Uric acid had maximum absorption at 292 nm. The absolute recovery of uric acid from the extraction was tested by adding a known concentration of the standard solution to a plasma sample. The recovery percentage of uric acid was determined according to the equation: C1/C2 + N × 100, where C1 is the concentration of uric acid in the enriched plasma, C2 is the concentration of uric acid in the blank plasma, and N is the nominal concentration that was added to the plasma.

Allantoin quantification was performed as described before (16), with minor modifications. Allantoin was extracted from a sample of 300 µL of plasma, mixing with 600 µL of cold acetonitrile. The sample was centrifuged at 22,000 *g* for 15 min at 4°C and the sediment was re-extracted with 600 µL cold acetonitrile/10 mM phosphate buffer, pH 7.4 (1:1). The combined supernatants were immediately applied to an AG 1-X8 anion-exchange resin column. The purified sample was dried under nitrogen atmosphere at 60°C. The resulting residue was dissolved in 200 µL of 0.1 M NaOH and heated in boiling water bath for 20 min to hydrolyze allantoin to allantoate. Then, 300 µL of 1.5 mM 2.4-dinitrophenylhydrazine/2.5 M HCl was added for derivatization and subsequent incubation at 50°C for 50 min. The reaction mixture was dried, filtered, and injected (40 µL) onto a C18(2) Luna (25 × 4.6 mm, 5 µm particle size) column (Phenomenex, USA). The mobile phase constituted of 8.3 mM phosphate buffer in 5% acetonitrile, pH 6.1 (solvent A) and 8.3 mM phosphate buffer in 50% acetonitrile, pH 6.1 (solvent B), with a constant flow of 0.5 mL/min. The following gradient was used: 0.01 to 10 min of 5% solvent B; 10–12 min of 60% solvent B; 12–30 min of 100% solvent B; 30–32 min of 5% solvent B. The column was equilibrated for 10 min with 5% solvent B. Derivatized allantoin was detected at 360 nm. The quantification was obtained by plotting the peak area against a standard curve of allantoin (0.5–10 µM).

### Myeloperoxidase assay

Myeloperoxidase concentration was measured by sandwich enzyme-linked immunosorbent assay (ELISA) (Abcam, UK). Briefly, sample proteins were quantified and diluted to normalize them before reaction. Myeloperoxidase was captured in a 96-well plate impregnated with mouse monoclonal antibody. After incubation, a polyclonal antibody was added and the excess was washed with phosphate-buffered saline. Avidin-biotin peroxidase complex was added and the excess was washed with phosphate-buffered saline. The peroxidase substrate tetramethylbenzidine was added to reveal avidin-biotin binding. Myeloperoxidase concentration was detected at 450 nm and compared against a standard curve.

### Biochemical measurements and physical parameters

Body mass index was obtained by dividing the weight (kg) by the square of height (m) (3). Plasma glucose was measured by enzymatic hexokinase method using the ADVIA 1200 Siemens analyzer (Siemens, USA). Glycosylated hemoglobin (HbA_1c_) was measured by HPLC using the Bio-Rad Variant II HbA1C analyzer (Hercules, USA). Low density lipoprotein (LDL)-cholesterol and high density lipoprotein (HDL)-cholesterol were determined by a homogeneous colorimetric method without precipitation. Hypertension was defined as the use of medications to treat hypertension, systolic blood pressure ≥140 mmHg or diastolic blood pressure ≥90 mmHg. Diabetes was defined as having a medical history of diabetes, use of medications to treat diabetes, a fasting glucose ≥126mg/dL, glycated hemoglobin (HbA1C) levels ≥6.5% or a 2-h oral glucose tolerance test ≥200 mg/dL (17).

### c-IMT measurements

The technique for c-IMTmeasurement has been previously applied in population-based studies including the ELSA-Brasil (18). The protocol was performed using a Toshiba ultrasound system (Aplio XG™, USA) with a 7.5 MHz linear transducer. c-IMT was measured in the outer wall of a pre-defined carotid segment of 1 cm in length from 1 cm below carotid bifurcation during three cardiac cycles. The carotid images during three cardiac cycles were obtained and sent to the centralized reading center in São Paulo. We used MIA™ software to standardize the reading and interpretation of carotid scans as previously described. c-IMT measurements are reported as the maximum values for the thickness of the right and left arteries measured at the far wall (17).

### Statistical analysis

All continuous variables are reported as means±SD. Categorical variables are reported as numbers and proportions. Comparison between the two groups (c-IMT P≥75 and P<75) was performed by unpaired *t*-test. Associations were evaluated using multiple linear regression, analysis of variance (ANOVA). Statistical analyses were performed using SPSS version 17 software (SPSS Inc., USA) or GraphPad Prism 5.0. Results were considered statistically significant when P was <0.05.

## Results

Individuals in this study were all male, 45 to 60 years old, and with c-IMT lower than 1 mm to ensure analysis of an early process of atherosclerosis. Table 1 compares the demographic and clinical characteristics according to c-IMT ≥P75 and c-IMT <P75 groups. The parameters race, use of anti-hypertensive drugs, and diabetes were equally distributed in the two groups. Traditional markers such as LDL, HDL, and BMI, and two potential novel markers, neck circumference and pulse pressure (17), were very similar among groups.

**Table 1.** Demographic and clinical characteristics of the individuals involved in the study.

Characteristics	c-IMT ≥P75 (n=35)	c-IMT <P75 (n=35)
c-IMT mm, mean (range)	0.75 (0.61–1.02)	0.55 (0.25–0.7)
Age, years (mean)	51.9	48.8
Race/ethnicity (n, %)		
White (n=41)	19 (54.3)	22 (62.9)
Mixed (n=15)	8 (22.9)	7 (20)
Black (n=10)	6 (17.1)	4 (11.4)
Asian (n=4)	2 (5.7)	2 (5.7)
Body mass index (kg/m^2^)	25.8±0.6	24.6±0.5
Under anti-hypertensive medicine (n, %)	10 (28.6)	5 (14.3)
Diabetes (n, %)	10 (28.6)	7 (20)
Use of lipid-lowering agents (n, %)	4 (11.4)	2 (5.7)
Fasting plasma glucose (mg/dL)	122±52.1	110.3±10.1
HbA_1c_ (%)	5.8±1.5	5.4±0.6
LDL-cholesterol (mg/dL)	136.8±38.9	135±36
HDL-cholesterol (mg/dL)	55.5±15.0	51.4±13.6
LDL/HDL (mg/dL)	2.6±0.9	2.8±0.92
Neck circumference (cm)	38.4±2.6	37.7±2.2
Pulse pressure (mmHg)	44.24±8.3	44.6±7.9
MPO (ng/mL)	31.09±16.11	28.17±14.0
Uric acid (µmol/L)	296.27±91.21	232.99±75.43*
Allantoin (µmol/L)	4.11±2.55	2.75±2.06*

Plasma measurements are reported as means±SD. *P<0.05 by unpaired *t*-test. c-IMT: carotid intima-media-thickness; HbA_1c_: glycosylated hemoglobin A; LDL: low-density lipoprotein; HDL: high-density lipoprotein; MPO: myeloperoxidase.

A significant increase in the levels of uric acid (P=0.002) and allantoin (P=0.025) was found in the c-IMT ≥P75 group (Table 1). Therefore, we next performed a multiple linear regression to evaluate the association between uric acid an allantoin with c-IMT. The analysis was adjusted by age, by the traditional risk factor LDL/HDL ratio, by the non-traditional risk factors pulse pressure and neck circumference, and by the inflammatory marker myeloperoxidase. The multiple linear regression revealed a significant association of c-IMT with uric acid and an even stronger association with allantoin. Of relevance, this positive association was independent of traditional and non-traditional risk factors of CVD (Table 2). It is important to highlight that the aim of this study was to evaluate a very initial process of atherogenesis and, therefore, the individuals of the study were apparently healthy, within normal BMI, LDL, HDL, and fasting glucose levels.

**Table 2.** Multiple linear regression between c-IMT and different parameters.

Parameter	B	95%CI	P
Age	0.011	0.005 to 0.017	<0.001*
LDL/HDL	0.013	−0.018 to 0.044	0.397
Neck circumference	0.008	−0.005 to 0.020	0.095
Pulse pressure	−0.002	−0.006 to 0.001	0.112
MPO	0.000	0.001 to −0.002	0.803
Uric acid	0.0004	0.0001 to 0.001	0.014*
Allantoin	0.018	0.005 to 0.031	0.008*

Data are reported as the regression coefficient (B) and 95%CI. *P<0.05. Each parameter was adjusted against all the other parameters presented in this table. c-IMT: carotid intima-media-thickness; LDL: low-density lipoprotein; HDL: high-density lipoprotein; MPO: myeloperoxidase.

## Discussion

The association of serum uric acid with CVD, including subclinical atherosclerosis, has been demonstrated elsewhere (1,3–5,19). However, debate continues whether uric acid has indeed a causal role and could, therefore, be an independent factor to CVD. For instance, three independent studies showed that serum uric acid was associated with subclinical atherosclerosis (1,4,19), but a Mendelian randomization analysis showed that the causal effect of uric acid could be inflated by hidden pleiotropy (6). In the present study, the association of uric acid and its oxidation product allantoin was independent of LDL/HDL ratio, pulse pressure, and neck circumference. As previously reported, pulse pressure and neck circumference have presented a higher impact on c-IMT than traditional risk factors, including lipoproteins (17).

**Figure 1.**
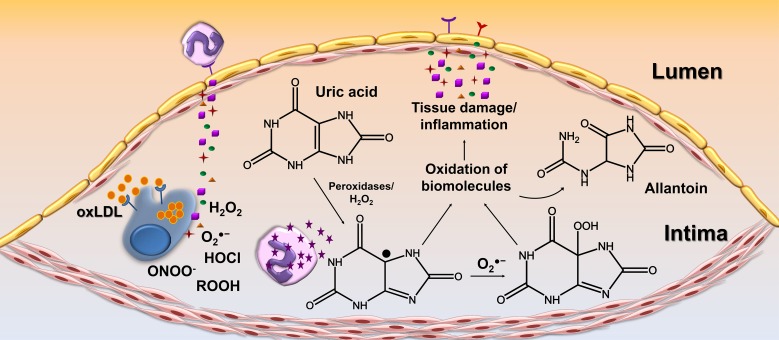
Uric acid reacts with oxidants from inflammatory cells in atheroma plaque. The oxidation of uric acid by hydrogen peroxide is catalyzed by inflammatory peroxidases, mainly myeloperoxidase, to generate urate free radical. The combination of this free radical with superoxide (O_2_^•−^) yields urate hydroperoxide. Both urate free radical and urate hydroperoxide are much stronger oxidants than hydrogen peroxide and superoxide and can easily oxidize neighboring molecules increasing tissue damage and inflammation. Allantoin is the main end-product of these reactions and, thus, allantoin plasma levels are directly correlated with the oxidation of uric acid. Therefore, plasma allantoin is an indicative of the oxidative stress in the intima layer and could be useful in the prediction of the very initial process of atherosclerosis.

The dual anti- or pro-oxidant property of uric acid has generated discussions whether it is protective or harmful. Uric acid is considered the main antioxidant in human plasma (7). However, the purine metabolism during uric acid production by xanthine oxidase generates the free radical superoxide and hydrogen peroxide (20). In addition, the neutralization of reactive oxygen species by uric acid does not necessarily produce inert yields. For instance, the neutralization of hydrogen peroxide occurs by a peroxidase-catalyzed reaction and this produces extremely reactive species, including urate free radical and urate hydroperoxide (9,10). Therefore, the vascular oxidation of uric acid could contribute to tissue damage and to the inflammatory process (Figure 1). The main breakdown product of these reactive uric acid-derivative species is allantoin (9,10). Allantoin has been proposed as a biomarker for oxidative stress (11,16). The levels of this metabolite were augmented in patients with chronic renal failure (16) and a substantial amount of allantoin was found in atheroma plaque (12,13). Increased urinary excretion of allantoin was also associated with the degree of atherosclerosis in mice (14). In this study, we found a significant strong association between allantoin and c-IMT, showing that allantoin could be a relevant marker in the early process of vascular disease. In spite of allantoin being a true marker for uric acid oxidation and oxidative stress in inflammatory conditions, this is the first study, to our best knowledge, that correlates allantoin with subclinical atherosclerosis.

We could not detect any correlation between c-IMT with plasma myeloperoxidase. Even though myeloperoxidase is an important enzyme related to inflammation, cardiovascular disease, and uric acid oxidation (9), it is not the only peroxidase present in plasma and is likely not the solely responsible for uric acid oxidation in plasma.

In summary, the present study demonstrated that plasma uric acid and allantoin were significantly and independently correlated with common c-IMT. These results showed that oxidation of uric acid and production of allantoin was a key event in subclinical atherosclerosis. Therefore, the measurement of allantoin in plasma, along with other traditional factors, could emerge as a relevant marker in subclinical atherosclerosis.
